# Predictive Biomarkers of Oxaliplatin-Induced Peripheral Neurotoxicity

**DOI:** 10.3390/jpm11070669

**Published:** 2021-07-16

**Authors:** Roser Velasco, Montserrat Alemany, Macarena Villagrán, Andreas A. Argyriou

**Affiliations:** 1 Neurology Department, Neuro-Oncology Unit-IDIBELL, Hospital Universitari de Bellvitge-Institut Català d’Oncologia L’Hospitalet, 08907 Barcelona, Spain; malemany@bellvitgehospital.cat (M.A.); mac.vgarcia@gmail.com (M.V.); 2 Institute of Neurosciences, Department of Cell Biology, Physiology and Immunology, Universitat Autònoma de Barcelona, and Centro de Investigación Biomédica en Red sobre Enfermedades Neurodegenerativas (CIBERNED), 08193 Bellaterra, Spain; 3 Neurology Department, “Saint Andrew’s” State General Hospital of Patras, 26335 Patras, Greece; andargyriou@yahoo.gr

**Keywords:** neurotoxicity, oxaliplatin, chemotherapy-induced peripheral neuropathy, biomarker, genomics, neuropathy, FOLFOX, FOLFIRINOX, XELOX, gastrointestinal cancer

## Abstract

Oxaliplatin (OXA) is a platinum compound primarily used in the treatment of gastrointestinal cancer. OXA-induced peripheral neurotoxicity (OXAIPN) is the major non-hematological dose-limiting toxicity of OXA-based chemotherapy and includes acute transient neurotoxic effects that appear soon after OXA infusion, and chronic non-length dependent sensory neuronopathy symmetrically affecting both upper and lower limbs in a stocking-and-glove distribution. No effective strategy has been established to reverse or treat OXAIPN. Thus, it is necessary to early predict the occurrence of OXAIPN during treatment and possibly modify the OXA-based regimen in patients at high risk as an early diagnosis and intervention may slow down neuropathy progression. However, identifying which patients are more likely to develop OXAIPN is clinically challenging. Several objective and measurable early biomarkers for OXAIPN prediction have been described in recent years, becoming useful for informing clinical decisions about treatment. The purpose of this review is to critically review data on currently available or promising predictors of OXAIPN. Neurological monitoring, according to predictive factors for increased risk of OXAIPN, would allow clinicians to personalize treatment, by monitoring at-risk patients more closely and guide clinicians towards better counseling of patients about neurotoxicity effects of OXA.

## 1. Introduction

Oxaliplatin (OXA) is widely used for the treatment of gastrointestinal cancers including colorectal (CRC), gastric, and pancreatic cancer, both in the adjuvant and metastatic setting [1,2]. OXA-induced peripheral neurotoxicity (OXAIPN) is the major non-hematological cause for dose-reduction as also discontinuation of OXA-based chemotherapy and it is manifested with two clinically distinct forms. The acute, neuromyotonia-like syndrome, as a result of hyperexcited sensory and motor nerves, appears soon after OXA, is transient and usually completely reversible within hours or days [3]. Patients may also develop chronic sensory symmetrical symptoms, including tingling, numbness and pain in a ‘stocking/glove’ distribution developing during treatment, while up to 20% of patients can be severely affected to develop sensory ataxia and increased susceptibility to falls [4,5,6]. Five years after finishing chemotherapy, 25–30% of patients suffer from clinically significant chronic OXAIPN [3,7], without modification in this rate over 3–8 years [8,9,10,11]. Persistent OXAIPN is associated with psychological distress, depression and impaired quality of life in long-term gastrointestinal cancer survivors [8].

**Figure 1:**
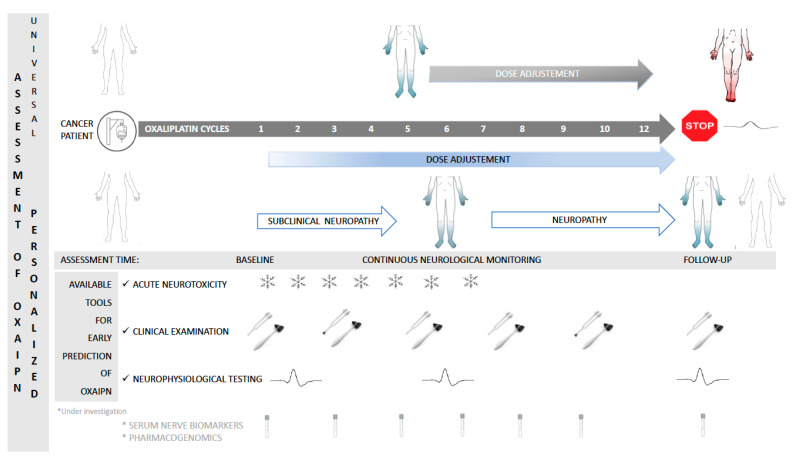
Personalized assessment of neuropathy by present and future strategies to early detecting and monitoring oxaliplatin-induced peripheral neuropathy in gastrointestinal cancer patients.

Given the lack of effective symptomatic or preventive treatment strategies against both acute and chronic OXAIPN [9], in daily practice, neurological symptoms referred by patients are usually taken into account to adapt OXA dosing in order to prevent severe neuropathy (Figure 1). Treating physician will indicate dose adjustment of OXA administering significantly less cumulative than planned doses of anti-cancer treatment what may compromise patient survival [10], therefore becoming a critical decision balancing the maximization of therapeutic benefit and the minimization of this significant OXA toxicity. Importantly, the observed worsening of OXAIPN weeks after cessation of treatment complicates clinical decisions regarding the duration and total dose of OXA in individual patients based on symptoms of sensory neuropathy alone. 

Cumulative dose of OXA is the main predictor of OXAIPN [12,13,14], with increasing rates from 42.5% to 76.7% with median cumulative dose of ≤780 mg/m^2^ and >780 mg/m^2^, respectively [11], also when re-challenging patients with OXA in further lines [15,16]. However, the relationship between dose and neurotoxicity might not be linear before reaching a cumulative dose level beyond which the toxicity becomes dose-dependent [17]. OXAIPN development cannot be accurately predicted in a gastrointestinal cancer patient before OXA treatment initiation and the common inter-individual variability in severity of OXAIPN in the setting of a uniform insult is a major challenge in clinical practice. Early prediction of development and progression of OXAIPN and a timely decision to decrease the OXA dosing in patients at high risk is clinically important. In recent years, several clinical and neurophysiological predictive biomarkers that can be easily obtained before or early during treatment to estimate which patients are at higher risk for OXAIPN have been described. From the personalized medicine perspective, having non-invasive, sensitive and specific biomarkers, will allow patients more liable to OXAIPN to prevent the occurrence of long-term toxicity or permanent damage. These objective markers may aid in the prediction of the development, severity and duration OXAIPN, and in adjusting OXA dose more precisely to balance the risk of neurotoxicity against antineoplastic efficacy. This review focuses on the currently available biomarkers of early OXAIPN detection that may allow clinicians to closely monitor at-risk patients and personalize treatment according to neurotoxicity risk of OXA.

## 2. Clinical Factors Associated with OXAIPN

### 2.1. Pre-Treatment Factors

A variety of pretreatment patient- or environmental-related risks have been described in the literature with conflicting results (Table 1). Methodological issues, such as the relatively small size, the retrospective design and the lack of statistical approach with multivariate regression analysis in many of them, likely underly limitations in the generalization of available results. To date, no demographic factors or preexisting comorbidities including age, diabetes mellitus or preexisting neuropathy have been consistently identified across multiple studies helpful in the prediction of the development, severity or duration of OXAIPN [18] that should be considered for upfront screening in a priori patients’ risk classification. 

### 2.2. Acute Neurotoxicity: The Main Clinical Predictor of OXAIPN

Shortly after every OXA infusion, many patients can experience cold-triggered painful paresthesia or neuromyotonia syndrome related with a transient axonal hyperexcitability of the peripheral nerve secondary to oxalate, generated after OXA biotransformation into its active form [3]. These acute symptoms are experienced by most of patients (90%) at some point of time during treatment [18,20,22], being usually reversible within hours or days and typically is triggered or exacerbated by cold [18,21,23,47] (Table 2). Similar to persistent OXAIPN, no factors beyond dose or infusion time seem related with the risk of developing acute OXAIPN [10,21,23,24,25,26,27,28].

Acute neurotoxicity is a well-established risk factor for developing chronic OXAIPN at the end of chemotherapy. Clinically, several studies show that the onset and severity of acute OXAIPN is associated with the occurrence of persistent neurotoxicity [4,10,20,24,29,30]. Attal et al. showed that the duration of cold-evoked pain and intensity of neuropathic symptoms experienced during the first three cycles predicted the extent of chronic pain experienced one year later. Cold-evoked symptoms lasting four days or more after 3rd OXA cycle predicted chronic OXAIPN (OR: 22; 95% CI: 1.54–314.74; *p* = 0.02) in a comprehensive comparative prospective study including 28 cancer patients receiving OXA, mostly CRC [20]. Our group identified that the burden of acute symptoms measured when patients have completed half of the planned OXA-based treatment (mid-treatment) was independently associated with nearly double risk (OR 1.9; CI 95% 1.2 to 3.2; *p* = 0.012) of developing severe chronic OXAIPN in a prospective multicenter study including 200 CRC patients [4,24]. In this line, presence of any acute neuropathy during cycles 1–3 was associated with persistent OXAIPN (HR: 3.65 (CI 95%; 1.40–9.56) *p* = 0.008) in a retrospective analysis of 50 CRC receiving FOLFOX schedule [12]. 

Early onset of acute OXAIPN seems particularly predictive of long-term neurotoxicity. Pachman et al. identified that patients who experienced severe acute OXAIPN within 6 days after first OXA infusion experienced more severe neuropathy in the remaining cycles and increased incidence of chronic OXAIPN (*p* < 0.001) [48]. Particularly, hyperacute neuropathy on the first day of the first OXA cycle was found to be a hallmark of risk of OXAIPN. Up to one third (27.7%) of patients developed hyperacute neuropathy in a retrospective study including 47 CRC patients receiving OXA-based chemotherapy. Of 13 patients who experienced hyperacute neuropathy, 12 (92.3%) eventually developed persistent OXAIPN. Multivariate analysis including the total dose of OXA and the presence of hyperacute neuropathy demonstrated that these two variables independently predicted OXAIPN [49]. The role of a such early clinical symptoms as further predictors require further validation in a in large multicenter study. 

Diverse strategies have been employed for assessing acute OXA neurotoxicity. The Common Terminology Criteria for Adverse Events from the National Cancer Institute (NCI.CTC) [10,44,50], the oxaliplatin-specific neurotoxicity scale [51], or a score based on recording the frequency of symptoms with an OXA-Neuropathy Questionnaire (yes/no response format) [17,52,53], are among the most common systems for recording their presence in the daily practice. The severity of acute neurotoxicity syndrome has been defined according the burden of symptoms [54], or according to a visual analogical scale 0 (no problem) to 10 (major problem) numerical rating scale for any of the four acute neuropathy symptoms [48,55,56]. More sophisticated techniques to objectively assess acute syndrome are described below in detail. 

Importantly, gastrointestinal cancer patients receiving OXA must be specifically interviewed about the presence of typical and atypical neuropathic symptoms, either these are transient or persistent. For example, recognition of acute atypical forms of OXAIPN requires a prolonged OXA infusion rate from 2 to 4 or 6 h in order to reduce risk of persistent OXAIPN [3,57]. The implementation of a simple standardized assessment tool to monitor acute neurotoxicity in daily clinical practice should be considered due to large amount of evidence supporting the predictive role of these early manifestations in predicting persistent OXAIPN. 

## 3. Neurophysiological and Device-Dependent Predictors

### 3.1. Nerve Conduction Studies (NCS) and Electromyography

Several longitudinal studies, including NCS during OXA therapy, have showed a significant progressive decrease in sensory nerve action potentials (SNAPs) and preservation of motor action compound (CMAPs), in keeping with the presence of an axonal sensory neuropathy, and consistent with the clinical symptoms and signs worsening during the treatment [17,41,58,59,60,61,62,63,64]. NCS are capable of objectively assessing the extent of peripheral nerve damage and may also facilitate the identification of patients that manifest subclinical peripheral neuropathy prior to the onset of clinically significant neurotoxicity. One cross-sectional study including 17 patients that had received a median of seven [8,45,65,66,67,68] treatment cycles and 850 mg/m^2^ at the time of testing, disclosed that almost half of patients had evidence of sensory neuropathy. After sensory examination, reductions in upper and lower limb SNAPs of patients were the most sensitive early markers of neuropathy observed in 40% [69]. In this line, in one prospective study including 60 gastrointestinal cancer patients, sural nerve velocity and SNAP revealed a significant decrease after 50% and 100% of the planned dose, respectively [60]. 

**Figure 2:**
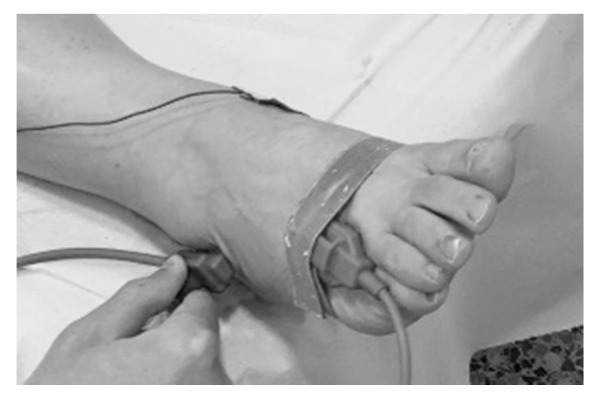
Nerve conduction study of the dorsal sural nerve.

NCS have also shown being useful in early predicting the neurological outcome at OXA completion. Early changes in the NCS results obtained during treatment were able to predict the development of severe OXAIPN in several prospective studies. A large multicenter study, including 200 CRC patients under treatment with FOLFOX-4, 6, and XELOX, identified at mid-treatment compared to baseline values a >30 % decrease in radial and dorsal sural SNAPs, while these abnormalities yielded a sensitivity and specificity of 96.3% and 79.1%, respectively, with positive and negative predictive values of 53% and 98.9%, for predicting severe OXAIPN at treatment completion. In the multivariate analysis, the three factors obtained at mid-treatment to independently and significantly be associated with an increased risk of severe neuropathy were: (1) having shown more symptoms of acute neurotoxicity (2) having a drop in the amplitude of the SNAP of the dorsal sural and radial nerves greater than 30%. The combination of these three factors allowed the patient with a high negative predictive value close to 99% to be classified a priori, so that in those patients with optimal NCS in the middle of treatment and who have not developed many symptoms of acute neuropathy, we could ensure with a high probability that he/she will not develop severe OXAIPN [17]. The predictiveness of dorsal sural nerve in risk stratification for OXAIPN was further evaluated in a secondary analysis of 100 CRC patients. An algorithm based on the dorsal sural nerve recordings showed that mid-treatment NCS could assign each patient to a ‘neurophysiological risk class’ for OXAIPN at the end of treatment [70]. In this line, reductions of the SNAPs of >11.5% in the median nerve between baseline and four cycles of OXA (odds ratio = 5.603, *p* = 0.031) and of >22.5% in the sural nerve between four and eight cycles of chemotherapy (odds ratio = 5.603, *p* = 0.031) were independently associated with the risk of developing severe OXAIPN [63]. However, very recently, negative results were obtained in another study evaluating the role of the sural nerve after administering 25% or 50% of the planned OXA dose in predicting the occurrence of clinically significant OXAIPN in 55 CRC patients [59]. The assessment of sural nerve instead dorsal sural (Figure 2), and the size of the study could underlie these negative findings. Long-term longitudinal neurophysiological assessments of OXA-treated patients have revealed a significant recovery of the SNAPs in sensory nerves in some studies [58,63,71] but not in other studies [59,61]. The length of follow-up observation may explain these differences. Unfortunately, to date, the correlation between SNAPs impairment and degree of neurotoxicity recovery remains unknown. In summary, growing evidence supports that NCS in distal sensory nerve segments offers clinicians a practical means of identifying patients more prone to severe chronic OXAIPN. 

Muscle sampling with needle electromyography (nEMG) can show repetitive myokymic discharges and neuromyotonic runs within 1–4 days after the first OXA administration [72,73,74]. However, the invasive nature of nEMG hampers its feasibility as screening tool to monitor gastrointestinal cancer patients undergoing OXA. Very recently, a simple painless objective tool to detect nerve hyperexcitability acute syndrome by a surface electromyography (sEMG) has been tested in a small study including CRC patients after the second (*n* = 14) and fourth (*n* = 8) OXA infusions revealing that sEMG is more sensitive (82%) than neurological examination (55%) to detect objective signs of acute neurotoxicity [55]. As such sEMG might be a promising test to evaluate acute oxaliplatin-induced motor nerve hyperexcitability and warrants to be further investigated in future studies.

### 3.2. Quantitative Sensory Tests (QST) 

Quantitative sensory testing (QST) examines subjective sensory function by measuring the abnormal detection and pain thresholds to several sensory modalities. The usefulness of these non-invasive psychophysical measurements in the clinic setting for detecting subclinical neurologic changes early on to identify patients that will experience OXAIPN has been largely explored [12,20,20,42,51,53,54,56,75,76,77,78,79,80,81,82].

Vibration sensation testing can be performed by a computer controlled vibrometer or, more easily, with a tuning fork placed on a bony prominence, such as the hallux or malleollus. In both, the subject reports when they can no longer detect vibration. Impairment of the vibration detection threshold (VDT) is generally seen over the treatment course [13,20,58,61] in correlation with the progressive loss of large myelinated fibers. However, conflicting results regarding the predictive role of early changes in VDT are seen in the literature. Whereas VDT in 30 patients with gastrointestinal malignancies receiving OXA evaluated at baseline and during infusion cycles 1, 2, 4, and 6, showed no clear relationship with OXAIPN development [80], two studies including 17 [69] and 60 patients [60] showed being the earliest marker of neuropathy, present at low cumulative doses of OXA. Significant change in VDT were present after the 25% of the planed dose, being the earliest among other measures [60]. Very recently, Kroigard et al. identified VDT measure obtained before treatment correlated as a predictor of clinically significant OXAIPN six months post-treatment. However, sensitivity of a baseline VDT < 5 (maximum 8) for the prediction of clinically significant neuropathy six months after treatment was modest (76.0% (95% CI 54.9% to 90.6%)) and specificity was low (53.3% (95% CI 34.3% to 71.7%)) [59].

Mechanical detection thresholds (MDT) measures have also shown significant deterioration with increasing OXA doses in some studies [42,60] but not in others [20,82]. Touch threshold changes became statistically significant in the fingertips at middle and 8 chemotherapy doses [42] whereas these findings only occurred after treatment completion in Kroigard’s study [60]. Very recently, electronic von Frey anaesthesiometer, which evaluates hyperalgesia based upon mechanical pain thresholds (MDTs), was tested prospectively in 46 CRC patients treated with OXA, and showed a decrease of 40 g in the MDT of both hands and feet as cut-off for diagnosing grade 2 or 3 OXAIPN with a total accuracy of 84.2% and 81.6% in hands and feet, respectively [78]. Besides diagnostic utility, the role MDT changes for predicting OXAIPN requires further research. 

Thermal detection thresholds have been particularly investigated due to the cold-induced nature of early acute OXAIPN [79]. Attal et al. was able to detect sustained signs of neurotoxicity at an early stage when the clinical manifestations appeared to revert between OXA cycles in a comparative prospective study including 48, mainly CRC patients with who were evaluated before OXA (*n* = 28) or cisplatin (*n* = 20) and after cycles 3, 6 and 9 and after completion. Enhanced pain in response to cold (20 °C stimulus on the hand) predicted severe neuropathy (OR: 39; 95% CI: 1.8–817.8 *p* = 0.02) [20]. Early changes in cold (CDT) and heat detection thresholds (HDT), as predictors of clinically significant neuropathic pain six months after treatment, has been recently identified by Kroigard et al. in a prospective study including 55 patients, 14 out of them with neuropathic pain. Reduced CDT after 25% of the planned OXA dose and reduced HPT after 50% of the planned dose measured at the dorsum of the right foot was correlated with neuropathic pain intensities. Change of −0.05 °C in CDT had a sensitivity and specificity of 92.3% (95% CI 64.0% to 99.8%) 64.9% (95% CI 47.5% to 79.8%), respectively, in predicting neuropathic pain six months after finishing OXAIPN. For change in HPT and the prediction of neuropathic pain, −0.85 °C had a sensitivity of 64.3% (95% CI 35.1–87.2%) and a specificity of 70.0% (95% CI 53.5–83.4%) [59]. Conversely, no association in cold and warm thresholds in 35 cancer patients treated with OXA-based regimen and OXAIPN was identified [82].

The role of QST to early identify OXAIPN remains vaguely defined. Among limitations, technically challenging methods of QST are not widely available, are time-consuming, and need standardized assessment algorithms and normative data which are not universally defined [83,84]. Besides, QST requires patient’s collaboration because results are based on a subjective response of the patient, compromising the objectivity desirable in a biomarker, and make QST not applicable in a subset of patients with impaired cognition and attention. Among QST parameters, VPT has the advantage of being quickly performed. Additionally, the equipment required is very portable and requires only basic training to operate. Accordingly, our and other authors experience [69] would favor VDT as the simplest and best routine marker among QST for detecting early OXAIPN in the clinic setting. 

Other devices to quantify tactile sensation (Bumps Detection test) [42,81] or small fiber (Sudoscan) [85] have been investigated for early diagnosis of large or small fiber impairments in subjects suspected of having OXAIPN, and for monitoring change over time, with promising albeit very preliminary results. Baseline deficits in sensory functioning measured using the Bumps Detection test were predictive of increasing numbness/tingling during the first 26 weeks of OXA-based chemotherapy [81]. Very recently, a multicenter study including 101 patients evaluated the usefulness of the CLIP test for early prediction of the risk of progression ≥grade 2 neuropathy in patients receiving chemotherapy with OXA. By testing the difficulty of patients in picking up and moving five gem clips one by one two squares and assessing patients experience in performing the test, authors identified that a positive result on the CLIP test (by asking patient to pick up and move a gem clip and whether there was some wrongness in doing it) was associated with an 8.3-fold higher risk of progression to ≥grade 2 OXAIPN. Noteworthy, a positive conversion of the CLIP test occurred before the progression to ≥grade 2 OXAIPN in 14 of the 21 (67%) patients [86]. The usefulness of this simple, cheap, and widely available assessment tool should be further validated in larger, multicenter prospective comparative studies. 

### 3.3. Axonal Excitability and Skin Biopsy

OXA produces acute changes in peripheral nerve excitability by modulating axonal voltage-gated Na^+^ channel activity [74,87]. Nerve excitability studies evaluate axonal excitability to provide information of the properties of the nerve membrane and of the ion channels expressed on these axons [88]. Acute symptoms after OXA infusion correlate with nerve excitability findings [56]. This method can assess acute OXA-induced abnormalities in sensory or motor nerve function [56], and its cold-triggered aggravation [89]. Measurement of excitability parameters have been consistently shown to be a sensitive early biomarker of ongoing OXAIPN, even preceding the reduction in the SNAP and development of symptoms [76,90,91]. Patients who demonstrated changes in excitability in early treatment, shortly after infusion, were subsequently more likely to develop moderate to severe neurotoxicity. Park et al. reported that an increase in the superexcitability of more than 15% prior to 5 of 12 chemotherapy cycles was able to identify 80% of patients with moderate or severe chronic OXAIPN. Acute changes in axonal excitability parameters that developed in early treatment cycles anticipated development of later neurotoxicity in patients who completed seven or more treatment cycles. Patients who completed treatment with moderate to severe neurotoxicity showed greater changes in early treatment (cycles 1 or 2), particularly reductions in the associated hyperexcitability [76]. Despite OXA causes acute excitability changes in both motor and sensory axons, progressive cumulative changes were only found in sensory nerves, and motor nerve excitability studies did not reveal early cumulative changes following treatment with OXA [69,76]. 

Nerve excitability testing provides a sodium channel dysfunction index and an objective biomarker of acute OXA neurotoxicity useful to improve prediction and risk stratification for OXAIPN prior to the onset of chronic neuropathy [56]. However, their scarce availability in most of centers, time-consuming nature and the lack of standardization for the clinical testing [88], converts this technique in too complex for routine screening, and not applicable in daily clinical practice for early detection of OXAIPN.

Skin biopsy is a minimally invasive method to evaluate neuropathy, especially small fiber nerve damage. Five prospective studies have incorporated skin biopsy in assessing ongoing OXAIPN. Contradictory results on the change over time of intraepidermal nerve fiber (IEFN) are available [59,60,64,92,93]. No significant early reduction in IENF during OXA treatment has been demonstrated; evidence which could be related with the fact that loss of IENF, a marker of axonal degeneration, is usually a later event occurring in peripheral nerves [60,84]. Besides, ongoing regeneration of small nerve fibers during OXA could contribute to these discrepancies [59]. Therefore, skin biopsy should not be used for predicting OXAIPN.

## 4. Pharmacogenomic Biomarkers

**Figure 3:**
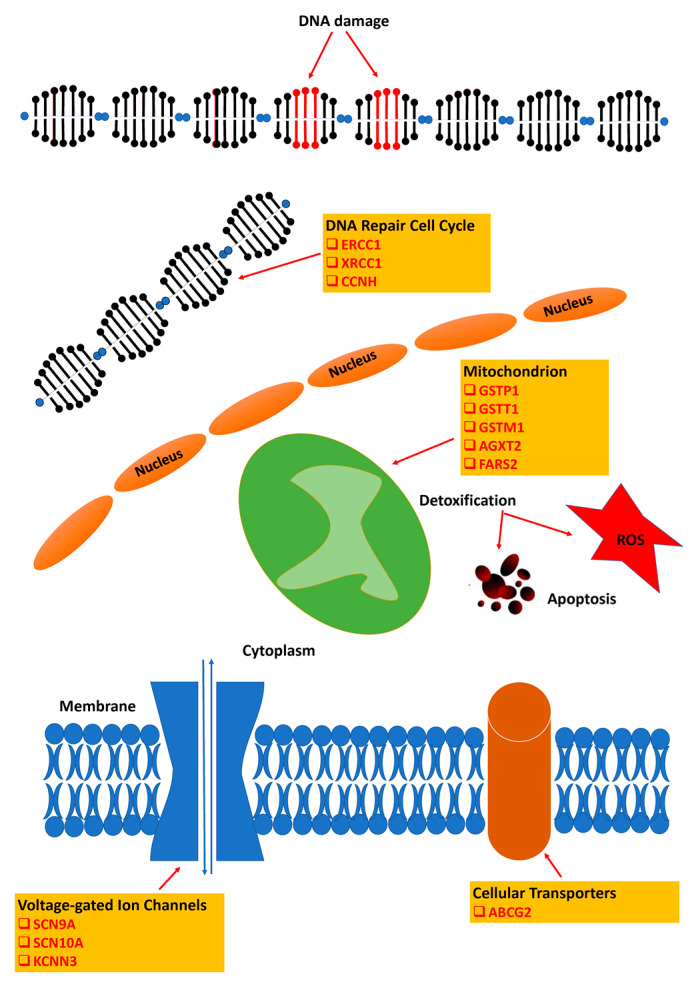
Cellular processes and main candidate genes implicated in oxaliplatin-induced neurotoxicity.

Genetic factors may contribute to a patient’s risk of experiencing OXAIPN. Over the last years, the development of pharmacogenetics, used to characterize human genetic variation, facilitated extensive efforts to understand the genetic basis of OXAIPN and to identify a specific genetic profile that can identify patients who are more liable to severe chronic neurotoxicity at the end of treatment. The majority of published studies assessed individual OXAIPN susceptibilities on single nucleotide polymorphisms (SNPs), which are mainly associated with gene variations in detoxification enzymes; DNA repair; drug transport; metabolism; neuronal receptors and ion channels (Figure 3). Furthermore, other genome wide analysis studies (GWAS) attempted to identify and validate SNPs mainly in genes encoding proteins implicated to neuronal function [94,95].

Some of these studies are of interest; tellingly, however, they have provided inconsistent findings and failed, in principle, to be replicated by other independent groups because of significant methodological limitations, including small sample sizes; retrospective study design; implementation of a post hoc analysis of oncology-based databases of different, not pre-planned size; lack of a pre-study hypothesis based on the known role of the investigated targets in the peripheral nervous system; inappropriate outcome measures for neurological impairment and differences related to DNA origin, extraction and genotyping [96].

### 4.1. SNP Studies

#### 4.1.1. Glutathione-S-Transferase P1 (GSTP1), Glutathione-S-Transferase T1 (GSTT1) and Glutathione-S-Transferase M1 (GSTM1) Genotyping

Genetic variants for GSTP1 exon 5 (Ile105Val), GSTP1 exon 6 (Ala114Val), GSTM1 (homozygous deletion), and GSTT1 (homozygous deletion) were examined in a cohort of 64 OXA-treated CRC patients, among whom 15 had grade 3 chronic OXAIPN. Patients homozygous for the GSTP1 105Ile allele more frequently encountered grade 3 OXAIPN compared to patients homozygous or heterozygous for the GSTP1 105Val allele (OR: 5.75; 95% CI: 1.08–30.74; *p* = 0.02). GSTM1, GSTT1, or GSTP1 exon 6 genotypes have not been associated with severe chronic OXAIPN [97]. In another study enrolling 63 OXA-treated (mFOLFOX6) metastatic CRC patients, it was shown that GSTP1–105 (*p* = 0.03) and GSTM1 (*p* = 0.02) were associated with increased incidence of severe chronic OXAIPN [98]. Another two studies disclosed a significantly reduced risk of OXAIPN with the GSTP1 AA genotype (Ile/Ile) [99,100], while the same increased risk of OXAIPN manifestation was reported with the GG (Val/Val) genotype [101,102]. Noteworthy, there are several reports with controversial results showing no association of GSTP1 Ile105Val with increased incidence of OXAIPN [103,104,105].

#### 4.1.2. ATP-Binding Cassette Transporter 2 (ABCG2) Genotyping

Custodio et al. performed genotyping in a cohort of 206 stage II-III OXA-treated CRC patients and a validation set of another 181 patients. Significant associations emerged for the CCNH rs2230641 C/C (OR: 5.03, 95% CI: 1.061–2.41, *p* = 0.042) and the ABCG2 rs3114018 A/A alleles (OR:2.67; 95% CI: 0.95–4.41; *p* = 0.059) with higher risk of grade 2–3 OXAIPN, while patients harboring the combination of these genotypes had significantly increased risk of severe OXAIPN, compared to patients carrying the CCNH any T and ABCG2 any C genotypes (37.73% vs. 19.42%; OR:2.46; 95% CI: 1.19–5.07; *p* = 0.014) [106]. 

However, replication of these results failed to achieve in a subsequent study enrolling 465 stage II or III CRC patients of Asian origin who were treated with the adjuvant-modified FOLFOX6 regimen. In the latter setting, comparison of low grade (0/1) OXAIPN with significant grade 2/3 OXAIPN did not showed any significant associations with any of the 12 examined SNP markers, including ABCG2 rs3114018 and CCNH rs2230641 [107]. In line with these negative results concerning the relevance of ABCG2 SNPs with OXAIPN, are the findings of a recently published study that tested germline DNA from 120 OXA-treated CRC patients together with a validation cohort of 80 patients and found no significant associations between ABCG2 (c.421 C > A/rs2231142) and increased incidence of OXAIPN [108]. 

#### 4.1.3. Cyclin-H (CCNH) Genotyping

The association of CCNH rs2230641 C/C with an increased incidence of severe OXAIPN has been demonstrated in the Custodio et al.’s study (2014), which was mentioned earlier [106]. Furthermore, the same effect of CCNH genotypes in acute OXAIPN was demonstrated in another study enrolling 228 OXA-treated digestive tract cancer patients. This study revealed that the CCNH-rs2230641 (AA vs. AG+GG; dominant model) and CCNH-rs3093816 (AA vs. AG+GG; dominant model) were both found significant for higher risk of more frequent and severe acute OXAIPN [109]. 

#### 4.1.4. X-ray Repair Cross-Complementing Protein 1 (XRCC) Genotyping

Genetic variants of the XRCC1 G/G polymorphism, which results in an Arg399Gln substitution has been tested for OXAIPN relevance in a study prospectively enrolling 292 Korean patients treated with FOLFOX for CRC and found that patients harboring XRCC1 23885GG experienced less grade 2–4 OXAIPN (adjusted OR:0.52, 95% CI: 0.27–0.99) [110]. 

Nonetheless, many other studies reported concordant evidence for lack of relevance between XRCC1 SNPs and OXAIPN, including Arg399Gln substitution [106,111]; Arg194Trp [112]; Arg280His [106,112]; rs3213239 [112]; rs12611088 and rs3213255 [106]. Subsequently published studies further provided evidence in support of the absence of any relevance of XRCC1 variants to OXAIPN features [105,107]. Tellingly, however, in the Kanai et al. study (2016), the proportions of patients developing grade 2–3 OXAIPN was quite higher compared to the Ruzzo et al.’s study (2014) (40.2% vs. 25.5).

#### 4.1.5. Voltage-Gated Sodium Channels (SCNA) Genotyping

The SCN2A R19K polymorphism failed to be associated with liability to OXAIPN in a study in which 62 advanced CRC patients were genotyped [113]. Similarly, no significant association emerged between SCN9A variant rs6746030 and OXAIPN in a subsequent study comprising 200 CRC patients [114], contrasting the results of a smaller study in which SCN9A rs6746030 was protective of severe OXAIPN in a heterogeneous population of 94 patients with various digestive tract cancers, and an increased incidence of coexisting diabetes (24%) in patients with grade 3–4 OXAIPN [115]. Tellingly however, a subsequently published study performed genotyping in 228 Indian OXA-treated digestive tract cancer patients and found increased susceptibility to chronic OXAIPN with the rs6746030 polymorphic variant of SCN9A (GA+AA vs. GG: OR: 1.8; 95% CI:1.04–3.4; *p* = 0.04; dominant model), while the SCN10A polymorphic variant was associated with severity of chronic OXAIPN (OR:2.0; 95% CI:1.2–3.3; *p* = 0.006) [11]. 

Finally, in the Argyriou et al. study (2013), it was disclosed a significant association between the SCN4A variant rs2302237 and increased risk of any grade chronic OXAIPN (OR:2.47; 95% CI: 1.04–5.85; *p* = 0.037) and more severe acute OXAIPN (OR: 2.50; 95% CI:1.35–4.63; *p* = 0.0029) [114]. 

#### 4.1.6. Voltage-Gated Potassium Channels (KCCN3) Genotyping

Basso et al. (2011) provided evidence for a significant association between 13–14 CAG repeat allele of KCNN3 (SK3) gene and development of acute OXAIPN (OR with >15 repeats; 0.381, 85% CI: 0.247–0.590, *p* = 0.001) in a small cohort of 40 CRC patients [116]. Another study, enrolling 86 CRC patients, did not show a significant association between chronic OXAIPN and KCNN3 repeat polymorphism [117]; concurring with the same negative results of a larger study studying 151 CRC patients and which provided evidence for lack of significant association between variations of the KCNN3 repeat polymorphism and development of either acute or chronic OXAIPN [113]. In any case, several mechanistic insights in OXAIPN pathogenesis are not supportive for a significant and direct involvement of K+channels in OXAIPN manifestation [118].

### 4.2. GWAS Studies

Won et al. (2012) performed GWAS in a discovery set of 96 and a validation cohort of 247 OXA-treated CRC patients of Asian origin in order to identify potential genetic markers for severe OXAIPN. This study identified and validated nine SNPs in eight genes [119], that failed to replicate in an independent validation in Caucasian patients [120]. The different genetic background of patients in the original and replication GWAS might hold the main response for conflicting results. No association was found with SNPs in ERCC1, GSTP1, XRCC1 and SNCA1 [119]. Finally, a very recently published study, enrolling over 1000 patients treated with paclitaxel, paclitaxel plus carboplatin, or oxaliplatin reported significant associations between rs56360211 near PDE6C (*p* = 7.92 × 10^−8^) and rs113807868 near TMEM150C (*p* = 1.27 × 10^−8^) with peripheral neurotoxicity. Tellingly, however, these results emerged from a polled analysis of treated patients and genetic associations were not tailored according to different chemotherapy compounds delivered [121].

There is increasing evidence pointing to the role of pharmacogenetics and pharmacogenomics in neurotoxicity susceptibility to OXA. Whereas pharmacogenetic results are currently being used in clinical decision making to inform treatment regimen choice with agents such as anthracycline [122] or fluoropyrimidine [123], larger-scale and validation studies are needed to further identify susceptibility markers of OXAIPN and to develop pharmacogenomics based-risk profiling to improve quality of life of gastrointestinal cancer patients. 

## 5. Imaging Biomarkers 

Data on imaging biomarkers in OXA-treated patients are still very limited, despite the benefit of their non-invasive nature. Nerve size, estimated by cross-sectional area (CSA), measured by nerve high-resolution ultrasound (HRUS), is an imaging modality that allows a quantitative structural analysis of the nerves. Existing clinical data on the application of HRUS in early assessment of OXAIPN are restricted to two small-sample-size studies. In 2013, Briani et al. conducted a cross-sectional study exploring the use of nerve HRUS in a cohort of 15 oxaliplatin-treated patients. The results showed an increased in CSA at common entrapment sites in 9 out of 15 patients prior to clinical symptoms and neurophysiological changes. At the end, 13 of the 15 patients developed a sensory axonal neuropathy [124]. More recently, Pitarokoili et al. conducted a prospective study on 13 oxaliplatin treated patients confirming an increase in CSA at upper limb entrapment sites and figuring out a CSA enlargement in tibial and fibular nerves. These findings also appeared either prior to or simultaneously with clinical and neurophysiology OXAIPN detection. No correlation between nerve HRUS, clinical severity and neurophysiology abnormalities was detected [125]. Moreover, a cross-sectional study of magnetic resonance neurography (MRN) in OXAIPN reported a significant dorsal root ganglia (DRG) hypertrophy after evaluating 20 patients. This finding correlates with one of the major mechanisms described for the initiation of neurotoxicity induced by this agent: the accumulation of platinum-DNA adducts in DRG [3]. Further investigation is required to establish the role of MRN as predictor of OXAIPN. Corneal confocal microscopy (CCM) is an ophthalmic noninvasive imaging modality that provides information of small sensory fibers by direct observation and scanning of corneal innervation with high resolution and magnification. Two prospective studies in gastrointestinal cancer patients are available, with conflicting results. Campagnolo M et al. demonstrated a reduction in the number and density of the corneal fibers after chemotherapy completion in 15 oxaliplatin treated patients [126]. Conversely, another study using CCM in 13 patients with upper GI cancer, eight of them who received 3 cycles of OXA containing regimes, identified a significant increase in corneal nerve fibre length [127].

Besides neuroimaging, other imaging techniques have been explored as surrogate biomarkers of early neurotoxicity. Preliminary evidences of the analysis body composition by computed tomography (CT) in gastrointestinal cancer patients reveal the loss of lean body muscle [128] or sarcopenic obesity [29] were independently associated with the occurrence of OXAIPN, supporting its potential predicitive usefulness that deserves further investigation. Other radiologic measures including spleen volumetric analysis in CT scan [129] or muscle ultrasound [130] have been anecdotically explored in OXAIPN. To date, further larger research in imaging techniques is needed to provide further in-depth objective evidence in order to transfer them into daily clinical practice as predictive biomarkers of OXAIPN.

## 6. Blood Biomarkers

Several whole blood biomarkers have been investigated as predictive tools in the assessment of OXAIPN. Neurofilament light chain (NfL) is a neuron-specific cytoskeletal protein expressed in large-myelinated axons [131] released in blood when nerve damage occurs. Supported by a preclinical studies in vincristine-induced peripheral neuropathy [132], Kim et al. conducted a prospective study including 43 patients treated with OXA and measured serum NfLs during (at 3 months) and post-treatment (at 6 months). An increase in NfL levels in both periods was observed, being higher at 6 months of OXA-treatment. High serum NfL levels correlated with neuropathy severity, providing compelling evidence of NfLs as a potential predictive marker of OXAIPN. Interestingly, after 4–6 months of follow-up a meaningful reduction on NfL levels was observed, indicating that NfLs can also discriminate recovery patients from OXAIPN [133]. Despite promising, the predictive usefulness of NfL as a biomarker requires independent validation with further studies with larger sample sizes that allow researchers to establish universal reference values in order to maximize the correct interpretation of NfL in the management of OXAIPN [131]. 

Preclinical evidences showed a significant reduction in Nerve Growth Factor (NGF) levels during OXA administration in rat [134], which was correlated with the onset of peripheral neuropathy. Considering clinical studies, limited data is available. Velasco et al. conducted a prospective observational study including 60 cancer patients, of which 35 were OXA-treated with. The objective was to investigate the changes in circulating NGF levels and IENF in the presence or absence of neuropathic pain [93]. This research was based on the rationale that NGF receptor, TrKA, is located in the terminals of sensory neurons. Thus, the interaction between NGF and its receptor activates intracellular pathways affecting the sensitivity of nociceptors. The results of the study demonstrated an association between increased NGF levels and patients developing painful chemotherapy-induced peripheral neuropathy (CIPN), whereas NGF levels remained stable in patients with either painless or absent CIPN. Additionally, NGF level increases correlated with the severity of neuropathic pain reported by patients. However, no association between NGF levels and IENF was detected. Despite these promising results, previous studies did not report this association, therefore the role of NGF as a biomarker for the severity of painful OXAIPN remains unclear. 

Other parameters routinely available from whole blood have been reported potentially useful in predicting the development of OXAIPN (Table 1). Pretreatment low hemoglobin level or anemia has been identified as a risk prognostic factor for OXAIPN in several studies. Mizrahi et al. carried out a large cross-sectional study including 105 patients treated with OXA. The results showed a correlation between reduced pretreatment levels of hemoglobin, detected in 24.5% of the cohort, with a greater severity of neuropathy [14]. In addition, previous studies on patients receiving OXA concluded similar results [15,19,22,27]. At pathophysiological level, a plausible explanation relating low hemoglobin levels and chronic neurotoxicity development is still unknown. 

Among pretreatment metabolic and nutritional blood-based biomarkers, there are conflicting results. Such as higher albumin level [14] as hypoalbuminemia [15,19] have been associated with the risk of developing OXAIPN. Despite higher rates of neurotoxicity were described in patients with low levels of magnesium, the lack of evidence in this line [14] would support the known inefficacy of calcium-magnesium supplementation during OXA treatment as a neuroprotective approach [9]. Very recently and for the first time, higher serum gamma-glutamyl transferase (GGT) and a lower level of vitamin D have been identified as independent predictors of grade 2–3 OXAIPN in the multivariate analysis of a retrospective study including 186 gastrointestinal cancer patients [19]. Despite their easy collection and measure, non-invasiveness and objective interpretation of the results make blood tests perfect candidates to monitor neurotoxicity, more research is needed to better understand the pathophysiologic mechanisms underlying its role as prognostic biomarkers. 

## 7. Conclusions

Early detection of OXAIPN is essential in the prevention of irreversible nerve damage and should be prioritized when assessing and evaluating patients receiving OXA treatment so that adequate adjustment in scheduled treatment plan can be made. Early clinical and neurophysiological signs of OXAIPN can be observed after low doses of OXA. The assessment of acute neurotoxicity symptoms in the routine clinical evaluation is a reliable biomarker for predicting the occurrence and development of OXAIPN, and particularly, the first cycles can be very informative. Regarding neurological examination, conflicting evidence on the timing of the threshold impairments hamper their currently use to inform clinicians in the prophylaxis of neuropathy. The development of a clinical standardized prognostic neuropathy assessment tool in order to early detect neuropathy should be validated. Despite, currently not part of common oncology practice, neurological monitoring with NCS may provide valuable reliable metric data in accurately disclosing and following the course of OXAIPN over time, offering at the same time useful information for dose reduction or discontinuation of the treatment before the progression to severe OXAIPN. Additionally, screening methods incorporating pharmacogenetics, may help to predict OXAIPN on the basis of genetic susceptibility and, consequently, allow a better, more personalized treatment. The further identification and validation of the simplest, non-invasive reliable and valid blood biomarkers for the premature screening of OXAIPN to reduce the morbidity and impairment in the quality of life of patients with gastrointestinal cancers associated with chronic OXAIPN might be of particular interest in neuroprotection trials. In the next future, by combining clinical, neurophysiological, genetic and potentially serum-based risks, decision-making would be improved to optimize treatment and prevent potentially serious neurotoxicity. The best noninvasive and easy-to-perform objective method to early detect and follow OXAIPN progression in the daily clinical practice in the hospital setting warrants further investigation and validation in larger prospective studies.

## Figures and Tables

**Table 1:** Factors investigated that are or not associated with the incidence and severity of oxaliplatin induced peripheral neurotoxicity.

Variable at Baseline	Associated	Not Associated
Age	[12] * [14,15,19] * [13,16]	[20,21] [22,23] *
Gender	[24]	[12,15,22] * [20,23,25]
BSA (Body Surface Area)	[12] * [13]	[23,26]
BMI (Body Mass Index)	[27,28]	[12,14,19,29] *
Body Weight	[12] *	[19] * [26]
Concurrent treatments	[12,30] * [31]	[32]
Creatinine (renal disfunction)	[33]	[12,19] * [34,35,36]
Diabetes Mellitus	[28,37] [38] ^#^	[12,15] * [38] ^&^ [27,39]
GGT	[19] *	
Hemoglobin	[14,15,19] * [22] * [27]	
Histopathology	[19] *	
Level of Albumin	[14,15,19] *	
Magnesium	[15,40] * [27]	[14] *
Preexisting Neuropathy	[41,42]	[12] *
Race	[22] *	
Season (winter)	[13,43]	[12,19,44] *
Stage of the disease	[19,23] *	[12,19] *
Schedule of chemotherapy	[45,46] [23] *	[19,44] *
Vitamin D	[19] *	

* Studies including multivariate analysis. ^#^ Shorter time to develop OXAIPN ^&^ No differences in the incidence of OXAIPN.

**Table 2:** Acute symptoms or signs of OXA induced neurotoxicity.

ACUTE OXALIPLATIN- INDUCED NEUROTOXICITY
Cold-induced perioral paresthesia
Cold-induced pharyngolaryngeal dysesthesia
Shortness of breath
Difficulty swallowing
Laryngospasm
Muscle cramps
Jaw stiffness
Visible fasciculations
Voice changes
Ptosis
Ocular changes

## Data Availability

Data sharing not applicable.

## Abbreviations

ABCG2	ATP-binding cassette transporter 2
AGXT2	Alanine-Glyoxylate Aminotransferase 2
CCNH	Cyclin-H
CCM	Corneal confocal microscopy
CIPN	Chemotherapy-Induced Peripheral Neuropathy
CRC	Colorectal Cancer
CMAP	Motor action compound potential
CSA	Cross-sectional area
CT	Computed Tomography
DRG	Dorsal root ganglia
ERCC1	Excision Repair 1, Endonuclease Non-Catalytic Subunit
FARS:2	Phenylalanyl-tRNA synthase 2
FOLFOX	Folinic acid, 5-fluorouracil, and oxaliplatin
FOLFIRINOX	Fluorouracil leucovorin irinotecan oxaliplatin
GSTP1	Glutathione-S-transferase P1
GSTM1	Glutathione-S-transferase M1
GSTT1	Glutathione-S-transferase T1
GWAS	Genome wide analysis studies
HRUS	High-resolution ultrasound
IENF	Intraepidermal nerve fiber
KCCN3	Voltage-gated potassium channels
MRN	Magnetic resonance neurography
NCI-CTCAE	Common Terminology Criteria for Adverse Events from the National Cancer Institute
NCS	Nerve Conduction Studies
NGF	Nerve Growth Factor
NfL	Neurofilament light chain
OXA	Oxaliplatin
OXAIPN	Oxaliplatin-induced peripheral neurotoxicity
QST	Quantitative Sensory Testing
ROS	Reactive oxygen species
SCNA	Voltage-gated sodium channels
SNP	Single nucleotide polymorphism
SNAP	Sensory nerve action potential
XELOX	capecitabine and oxaliplatin
XRCC:1	X-ray repair cross-complementing protein 1
